# Leaky Integrate and Fire Neuron by Charge-Discharge Dynamics in Floating-Body MOSFET

**DOI:** 10.1038/s41598-017-07418-y

**Published:** 2017-08-15

**Authors:** Sangya Dutta, Vinay Kumar, Aditya Shukla, Nihar R. Mohapatra, Udayan Ganguly

**Affiliations:** 1 Department of Electrical Engineering, IIT Bombay, Mumbai, 400076 India; 2 Department of Electrical Engineering, IIT Gandhinagar, Gandhinagar, 382355 India

## Abstract

Neuro-biology inspired Spiking Neural Network (SNN) enables efficient learning and recognition tasks. To achieve a large scale network akin to biology, a power and area efficient electronic neuron is essential. Earlier, we had demonstrated an LIF neuron by a novel 4-terminal impact ionization based n+/p/n+ with an extended gate (gated-INPN) device by physics simulation. Excellent improvement in area and power compared to conventional analog circuit implementations was observed. In this paper, we propose and experimentally demonstrate a compact conventional 3-terminal partially depleted (PD) SOI- MOSFET (100 nm gate length) to replace the 4-terminal gated-INPN device. Impact ionization (II) induced floating body effect in SOI-MOSFET is used to capture LIF neuron behavior to demonstrate spiking frequency dependence on input. MHz operation enables attractive hardware acceleration compared to biology. Overall, conventional PD-SOI-CMOS technology enables very-large-scale-integration (VLSI) which is essential for biology scale (~10^11^ neuron based) large neural networks.

## Introduction

Spiking neural network (SNN) is an attempt to understand and mimic human brain functionalities – a key challenge of next generation computing. SNN demonstrates energy efficiency advantages over von-Neumann architecture for recognition and classification tasks^1^. To construct SNN in hardware, an efficient analog to the biological neuron is essential. Primarily, Si CMOS technology is used for analog implementation of electronic neurons. The dynamic nature of neuronal cell has been successfully captured by analog circuits^2–7^. Also, analog neuron circuits provide area and power benefit^5^ compared to the digital^1^ implementation. But the high neuronal density (10^11^ neurons in the human brain compared to 10^9^ transistors/chip) and connectivity (10^4^ neurons connected to each neuron compared to a typical fan out of 8 in CMOS) imposes two major constraints. First, individual components (e.g. neurons and synapses) must be highly area and power efficient. Second, the technology must be sufficiently matured to enable extreme integration of numerous (~10^11^) neuron. Recently, our group has proposed a highly power and area efficient neuron on impact ionization based n+/p/n+ diode (I-NPN) device with an extended gate driven by a small reset circuit in a *device simulations* study^8^. Excellent area (60x) and power improvement (5x) is demonstrated compared to previously reported analog circuits^8^. Further, *record* low bias (sub-0.2 V) impact ionization in I-NPN is also *experimentally* demonstrated by our group^9^. Gate-I-NPN requires unconventional process integration that is challenging for experimental realization and 4 terminals that involve layout and interconnection challenges. Hence, the neuronal function still remains to be *experimentally* demonstrated. In this paper, we propose to replace the *4-terminal*, *novel* gated I-NPN device with a *conventional 3-terminal*, *highly manufacturable* PD-SOI MOSFET. We experimentally demonstrate neuronal behavior based on the intrinsic charge dynamics of the device.

## Background

**Figure 1:**
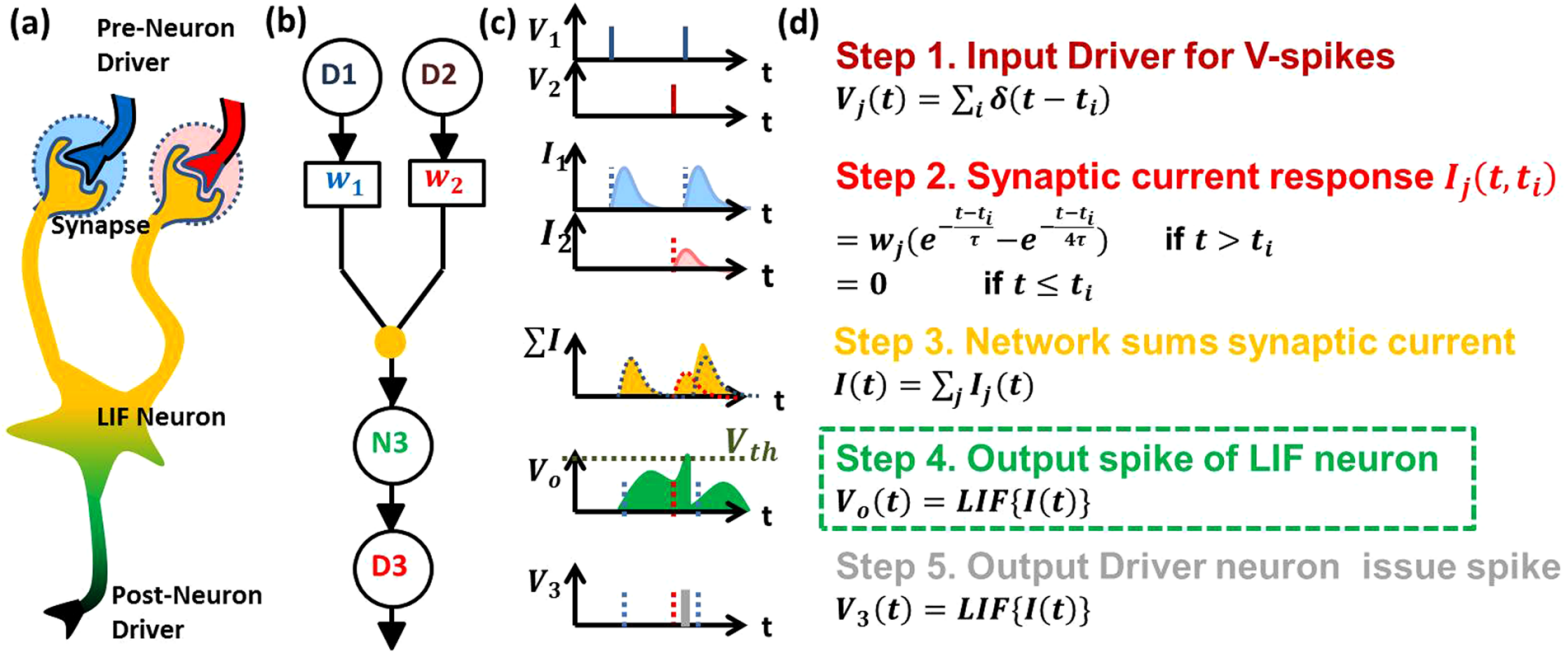
(**a**) Biological neuronal network is related to (**b**) algorithmic SNN analog. (**c**) The related signal timing (**d**) with step-wise signal evolution is shown. The SNN algorithm requires input spikes at times *t*_*i*_ from j^th^ pre-synaptic neuron driver e.g. D1, D2 (step 1). The spikes are converted into currents at synapse which depend upon synaptic strengths *w*_*j*_ (step 2). The currents are summed by the networks (step 3) and input into LIF neuron that determines neuronal firing instants (step 4) while post-neuronal driver (D3) produces spikes at the firing instants.

A simplified step-wise picture of SNN algorithm^10^ is shown in Fig. 1. First, pre-synaptic neuronal driver D1 and D2 provide the input voltage spikes (where i^th^ spike occurs at time *t* = *t*_*i*_). Second, these input spikes are converted to a gently varying current signal proportional to the synaptic weight (w_j_). Third, the current from synapses (w_1_and w_2_) is summed into the input of LIF neuron N3 by the network. Fourth, the LIF neuron (described in detail next) integrates the input current across a capacitor, which raises its potential. N3 resets immediately (i.e. loses stored charge) once the potential reaches/exceeds a threshold. Fifth, every time N3 reaches threshold, a driver neuron D3 produces a spike. The detail of this architecture is discussed in ref. 10. Among various neuronal models, the leaky integrate and fire (LIF) model can mimic the behavior of the biological neuron with minimum number of circuit element unlike other models^11–13^.

### LIF Neuron Model

**Figure 2:**
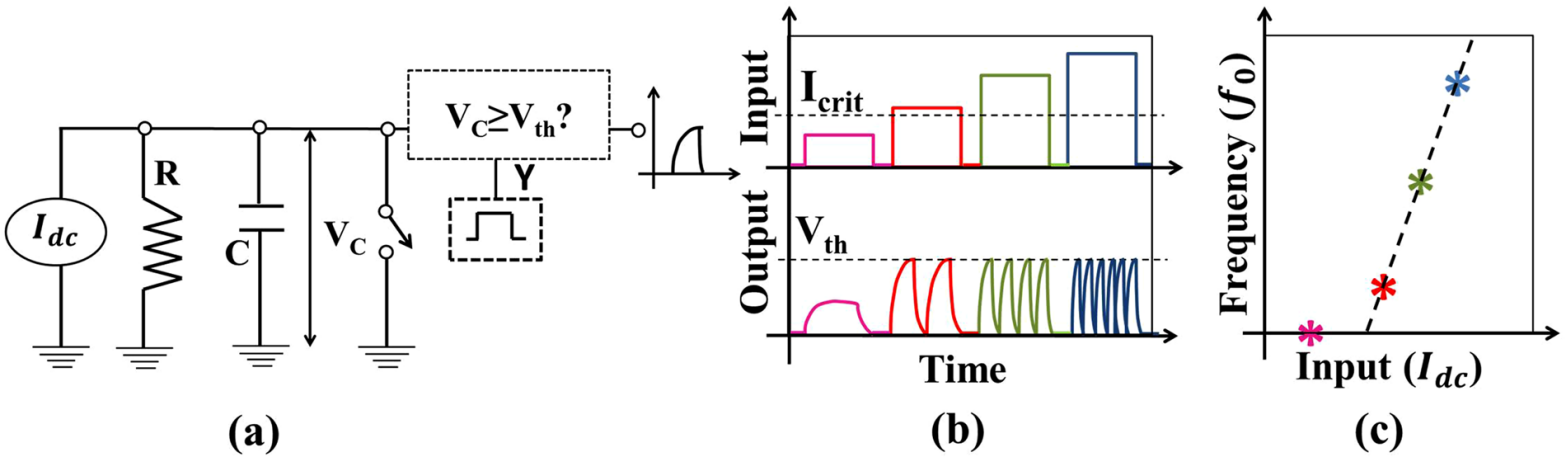
(**a**) LIF neuron circuit model, (**b**) For input (I_dc_ < I_crit_), V(t) never exceeds V_th_- hence neuron never spikes. However, for I_dc_ ≥ I_crit_, neuron will fire when V(t) ≥ V_th_ and immediately reset i.e. V(t) = E_L_, (**c**) With higher input (e.g. I_dc_ ≥ I_crit_), firing rate or the frequency increases like a biological neuron while for low input (I_dc_ < I_crit_), frequency is zero. The output frequency (f_o_) vs. input is the signature neuronal function to be mimicked artificially.

Leaky integrate and fire (LIF) model represents neuron as a parallel combination of a “leaky” resistor (conductance, *g*_*L*_) and a capacitor (*C*) as shown in Fig. 2(a). A current source *I*(*t*) is used as synaptic current input to charge up the capacitor to produce a potential *V*(*t*).

When potential exceeds threshold (*V*(*t*) ≥ *V*_*th*_), the capacitor discharges to a resting potential *E*_*L*_ using the voltage-controlled switch, like a biological neuron. Each time LIF neuron voltage exceeds threshold, a separate driver circuit (say, D3) issues a spike as mentioned in the last section. Thus, LIF model is governed by the following differential equation:

1 $$C\frac{dV}{dt}=-{g}_{L}(V(t)-{E}_{L})+I(t)$$

At low input current (*I*(*t*)), *V*(*t*) never exceeds threshold *V*_*th*_ - which produces no spikes (i.e. *t*_*i*_ → ∞). For example, if a dc input (i.e. *I*(*t*) = *I*_*dc*_) does not exceed a critical current (*I*_*crit*_ = *g*_*L*_(*V*_*th*_ − *E*_*L*_)), *V*(*t*) will always be less than *V*_*th*_ (i.e. *V*(*t*) < *V*_*th*_), by simple steady state analysis of Eqn. 1. When *I*(*t*) is high (e.g. *I*_*dc*_ > *I*_*crit*_), the charge up time to *V*_*th*_ reduces (Fig. 2b). This essentially increases the output frequency (*f*_*o*_) with increase in input *I*_*dc*_ (Fig. 2c).

### Device Structure, Concept and Operation

**Figure 3:**
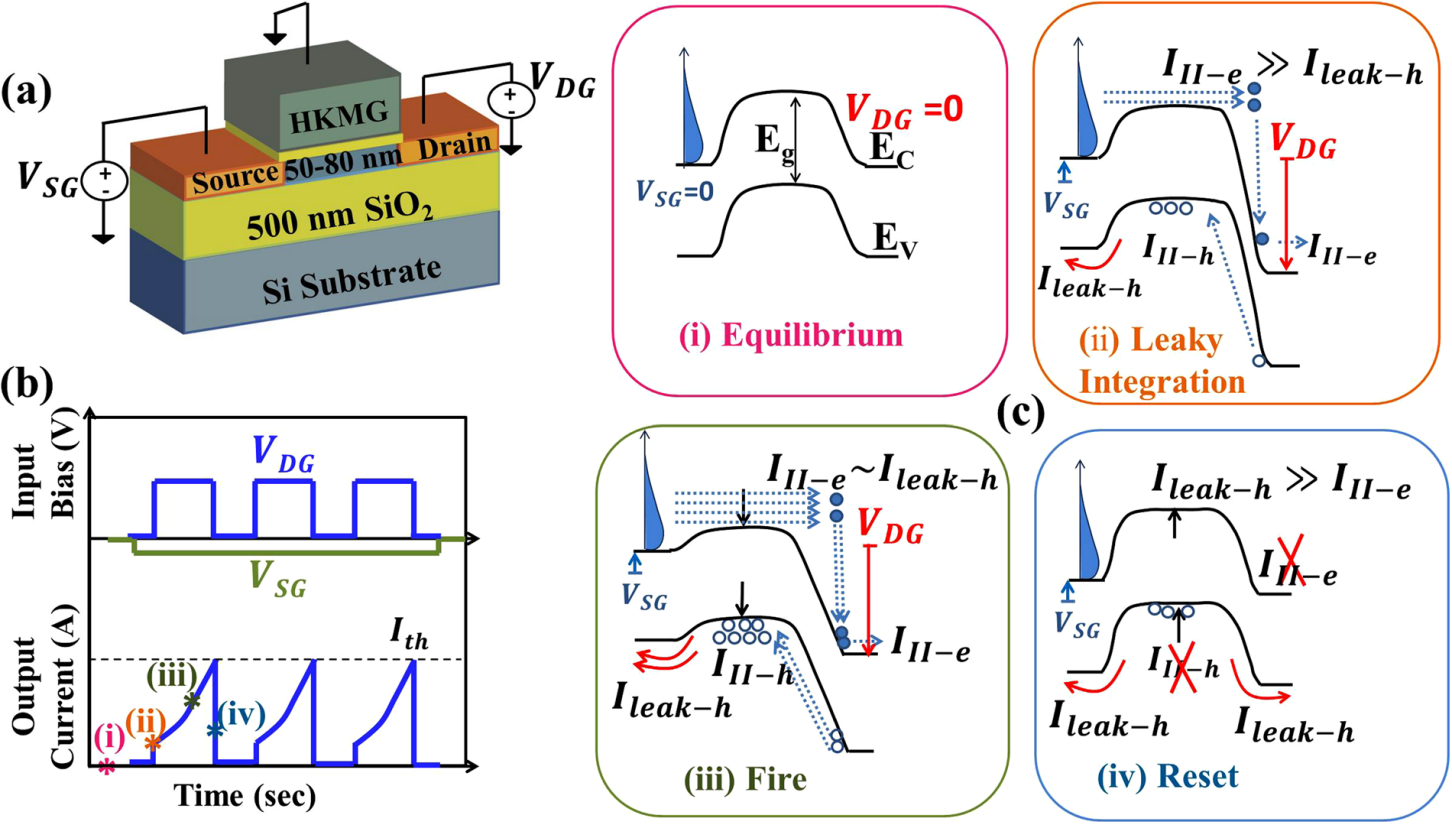
(**a**) Device schematic with biased terminals, (**b**) Input biasing scheme (V_SG_(t),V_DG_(t)) with expected output (I_D_(t)), (c-i) Equilibrium band diagram. (ii) Electron hole pair generation due to impact ionization (“integrate”) increases holes in well, (iii) Barrier lowering due to stored holes in the potential well. Also, the holes start to escape through the source junction (“leak”), (iv) Once threshold is reached (“fire”), removal of V_DG_ makes the holes escape through both the junctions bringing the barrier to its original position (“reset”). Thus, the charge dynamics enables the Leaky Integrate and Fire neuron in a PD-SOI n-MOSFET.

In this work, a simple PD-SOI MOSFET (schematic shown in Fig. 3a) is used to demonstrate LIF neuron like behavior. As in conventional CMOS, voltage input and current output are used. This is converse of biology. However, we focus on producing an input (*V*_*in*_(*t*)) vs. output frequency (f_o_) mapping (Fig. 2c). Voltage to current conversion and vice versa is trivial and maybe done based on system implementation needs. Figure 3b shows the biasing scheme where the input bias is applied on source (S) i.e. *V*_*SG*_(*t*) = *V*_*in*_(*t*). The drain (D) bias i.e. *V*_*DG*_(*t*) is used to select “integrate” vs. “reset” modes. Both *V*_*SG*_ and *V*_*DG*_ are referenced to a grounded gate (G). To explain the physics of operation, the output i.e. drain current (*I*_*D*_(*t*)) is annotated at four time instants (i–iv). At instant (i), the device is under equilibrium (Fig. 3(c-i)). At instant (ii), a low.

*V*_*SG*_ (~0.2 V) and a high *V*_*DG*_ (say 2.8 V to enable integrate) are applied to initiate impact ionization (II). The II generated electron (*e*) current (*I*_*II*−*e*_) escape through the drain while the hole (*h*) current (*I*_*II*−*h*_) flows into the channel potential well. Some fraction of *h*current leaks through the source barrier (*I*_*leak*−*h*_ i.e. equivalent to leaky integrate function in LIF) (Fig. 3(c-ii)). The net *h* current (*I*_*II*−*h*_ − *I*_*leak*−*h*_) builds up *h*-charge in the channel potential well over time (equivalent to integrate function in LIF). Increasing *h*-charge, electrostatically reduce the source *e*-injection barrier, allowing more electron injection for stronger II and set up a positive feedback (Fig. 3(c-iii)). The *e*-injection barrier reduction is coupled with *h*-well depth reduction which increases *I*_*leak*−*h*_. Steady state is achieved when *I*_*leak*−*h*_ balances *I*_*II*−*h*_ preventing additional *h*-storage. However, before steady state is reached, the current exceeds pre-set threshold (*I*_*th*_) (equivalent to fire function in LIF). The *I*(*t*) ≥ *I*_*th*_ condition sets off a small “reset circuit” that removes drain bias (*V*_*DG*_ = 0) disabling II (i.e. *I*_*II*−*h*_ → 0)^8^. Hence, all the stored holes leak (*I*_*leak*−*h*_) through both the source and the drain junction (Fig. 3(c-iv)) and resets the neuron back to the initial condition. After reset is complete, the drain is set back to high (*V*_*DG*_ = 2.8*V*) after a typical timescale (called “refractory period”) to re-initiate the LIF process (Fig. 3(c-ii)). Thus, the dc input *V*_*in*_(*t*) produces cycles of leakage, integration, firing and resetting (LIF process) to produce firing frequency (*f*_*o*_) in the output drain current (*I*_*D*_).

## Experimental Validation

**Figure 4:**
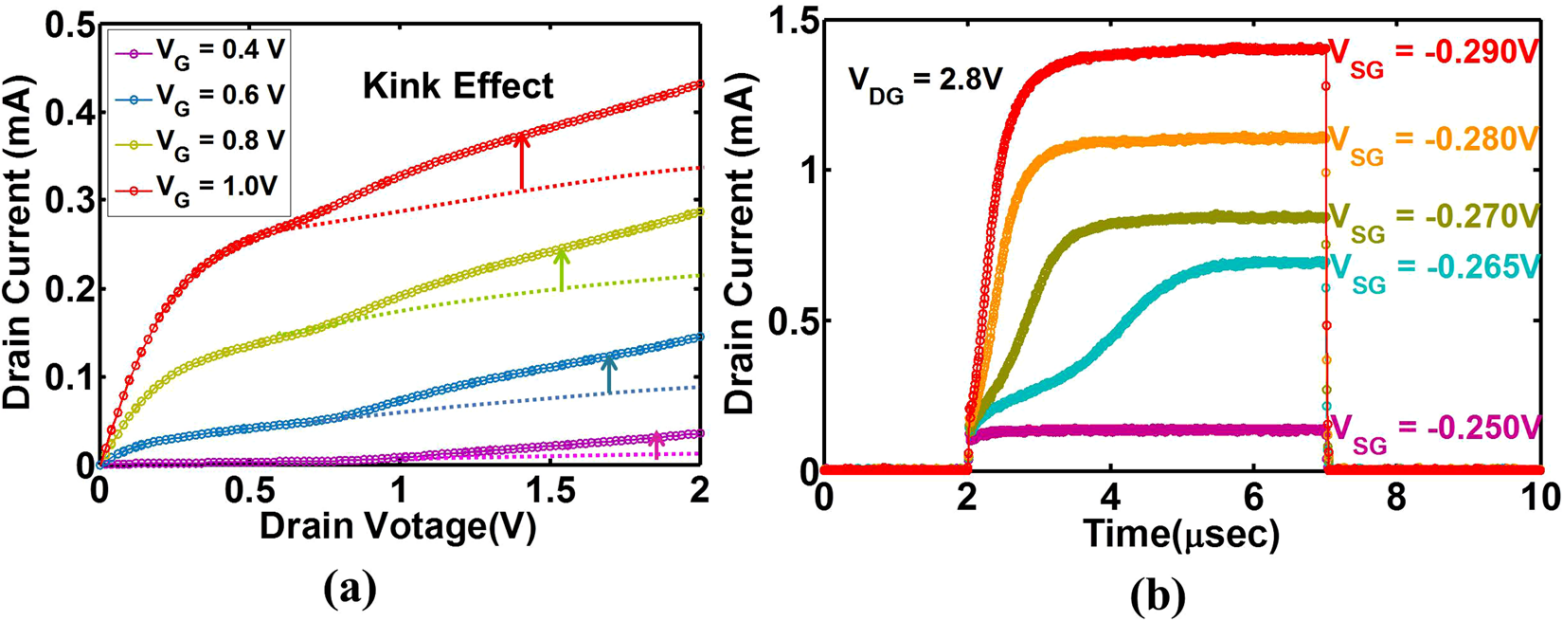
(**a**) I_D_ − V_D_ curve for different V_G_ shows “kink effect” as a signature of impact ionization induced floating body effect, (**b**) Transient measurement showing Leaky, Integrate functions for different input bias.

**Figure 5:**
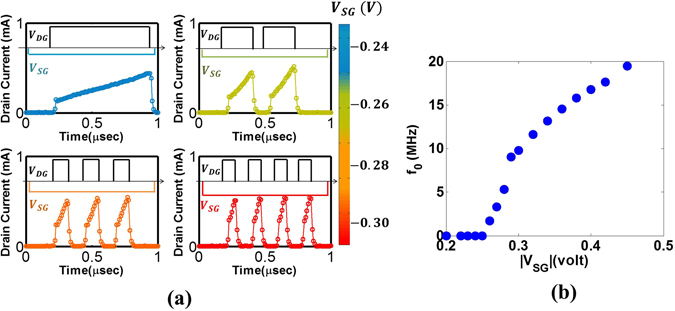
(**a**) The LIF function for different input (V_SG_) is shown with a V_DG_ based reset. At threshold (I_D_ ≥ I_th_ = 500 μA), V_DG_is grounded to reset the neuron for 100 ns, then set back to V_DG_ = 2.8 V. The output current starts from the same initial point after each reset – essentially make each LIF cycle identical. As V_SG_ is increased from (i–iv) more frequent fire & reset is observed which is akin to faster “spiking” with higher input bias (**b**) Output frequency vs. input shows the occurrence of input threshold i.e. |V_th_| = 0.26 V. If |V_in_| < |V_th_|, frequency is zero while for |V_in_| ≥ |V_th_|, f_o_ increases with input bias. Quantitatively, this device can provide 100000× higher frequency compared to a biological neuron, which typically fires at ~10 Hz.

The typical kink-effect is observed in the *I*_*D*_ − *V*_*D*_ (Fig. 4(a)) to confirm impact ionization^14^. Next, transient measurement shows the integration function of the LIF neuron where the applied source bias (*V*_*SG*_) represents input *V*_*in*_(*t*) (Fig. 4(b)). First, when only *V*_*SG*_ = −0.25*V* is applied (*V*_*DG*_ = 0), the device is still off i.e. current remains negligible. When *V*_*DG*_ = 2.8*V* is applied, an instantaneous increase in current is observed akin to Fig. 3(c-ii). A slower rise in current follows, as impact ionization produces a build-up of *h*-charge (i.e. integration). This, in turn, reduces the *e*-injection barrier to increase current to reach a steady state akin to Fig. 3(c-iii). We observe that the rate of current rise increases with input i.e. *V*_*SG*_. For lower *V*_*SG*_, the device is unable to initiate impact ionization due to lack of *e*-current supply. At high *V*_*SG*_, the rate of current rise increases and reaches steady state at a higher current (*I*_*D*_) level. To add the fire and reset, a current threshold *I*_*th*_ is set such that when *I*_*D*_ exceeds *I*_*th*_, we set *V*_*DG*_ = 0 manually. This can be automatically performed by an external circuit as explained earlier^8^. Figure 5(a) shows the reset effect where *V*_*DG*_ is set to zero if *I* ≥ *I*_*th*_ = 500 *μA* manually. For Fig. 5(a-i), *V*_*SG*_ = −0.24 *V*, threshold is achieved once in <1 *μs* duration followed by reset. For Fig. 5(a-ii–iv), *V*_*SG*_ is increased. The drain current rises faster (“integration”) to exceed threshold (i.e. *I*(*t*) > *I*_*th*_) followed by effective reset. Such cycles of integration and reset occurs naturally. We observe increasing frequency of reaching current threshold (fire) with increase in input *V*_*SG*_. Figure 5(b) shows output frequency vs. input *V*_*in*_ = *V*_*SG*_ akin to Fig. 2 (c). A threshold is observed such that |*V*_*in*_| < 0.26 *V* produces no spikes while above threshold, a linear dependence of spiking frequency (*f*_*o*_) on *V*_*in*_ is observed. This is the signature of LIF neuron. Further, this device offers higher frequency (in the range of MHz) compared to biology (~1–10 Hz), which enables attractive hardware acceleration^15^.

## Performance & Benchmarking (New Section)

To evaluate the area and power performance, we have implemented the neuron (i.e. SOI device with a reset circuit) to demonstrate spiking, evaluate energy per spike and estimate layout area (Supplementary Information 1). Further, we use 12 input and 3 output neurons based spiking neural network (SNN) for Fisher Iris classification to show state-of-the-art recognition (~95%) (Supplementary Information 2). Such an SNN algorithm has software-equivalent hardware implementation^21^. In Table 1, we benchmark SOI neuron with literature. First, the conventional current–driven CMOS analog implementations provide power benefit at the cost of large area consumption^5, 16, 17^. Novel voltage-driven neurons have been proposed^18–20^, though the materials used here are not standard CMOS compatible. Among several voltage-driven neurons, phase change memory (PCM) based neuron could be a power and area efficient counterpart to analog neurons. Integration of PCM *material* in an array has also been demonstrated^20^. Our proposed PD-SOI technology does not require any *new* materials integration. SOI technology is highly mature for VLSI implementation of numerous neurons. Additionally, SOI neuron has its capability of “leaky” integration without any extra circuitry unlike other neurons. The leakiness is an essential feature of biological neuron – which adds a time-dependent memory due to ion channel dynamics^22^. This is realized at the device level in SOI neuron owing to its inherent charge dynamics (i.e. recombination of excess carriers leakage through source/drain region). Also SOI neuron requires ~260 mV for firing compared to these novel neurons, which require higher threshold (>1 V)^18–20^. Our demonstration requires a smaller voltage swing (of the order of few hundred mV), which is closer to biological neuron (100 mV)^23^. This voltage range can be further reduced by SOI device engineering. The energy per spike is calculated to be 35 pJ for SOI device including the external circuitry, which is comparable to the phase change neuron (30 pJ only at the device level). However, phase change neuron requires digital implementation (including a global clock).Global clocks are power inefficient compared to asynchronous implementation. In fact, analog asynchronous implementations maybe as high as 10× more energy efficient^24^. Our implementation is asynchronous, which is energy efficient at the systems level and closer to biology.

**Table 1:** Benchmarking with state-of-the-art electronic neurons.

References	Neuron Type	Synaptic Input Type	Device Type	Circuit Type	Tech. Node	Area (*μ*m^2^)	*Vth*(*V*)	Energy/spike (pJ)
Giacomo Indiveri *et al*.^16^	**LIF**	Current	CMOS	**Analog-Digital**	0.35 *µ*m	2573 (~21 × 10^3^ *F* ^2^)	—	900
Jayawan H.B. Wijekoon *et al*.^17^	**LIF**	Current	CMOS	**Analog**	0.35 *µ*m	2800 (~23 × 10^3^ *F* ^2^)	—	**8**.**5–9**
A. Joubert *et al*.^5^	**LIF**	Current	CMOS	Digital	65 nm	538 (~127 × 10^3^ *F* ^2^)	—	41.3
Kibong Moon *et al*.^18^	IF	Voltage	IMT	—	—	—	1.3	—
Jaesung Park *et al*.^19^	IF	Voltage	IMT	—	—	—	1.6	—
Tomas Tuma *et al*.^20^	IF	Voltage	Phase Change	**Analog-Digital**	14 nm	**0**.**5–1** (2551–5102***F*** ^2^)	>1	**30**
**This work**	**LIF**	Voltage	**SOI MOSFET**	**Analog**	**32 nm**	**1**.**8** (1767F^2^)	**0**.**26**	**35**

## Conclusion

To summarize, a highly manufacturable Si based SOI-MOSFET is experimentally shown to demonstrate LIF neuron functionality. Intrinsic carrier dynamics of the device produces “Leak Integrate and Fire” functionality. This experimentally validated approach is noted for significant area and power efficiency compared to analog circuit implementation (Supplementary Information 1). By modeling the output characteristics of the SOI neuron, a MATLAB based spiking neuron network is shown to perform classification task with reasonable accuracy (~95%) (Supplementary Information 2).Also, this device offers higher frequency (in the order of MHz), than a biological neuron (~1–10Hz) to enable attractive hardware acceleration. CMOS based 32 nm SOI technology provides excellent maturity and very large scale integration (VLSI), which is essential for biology equivalent large scale spiking neural networks.

## Method

**Figure 6:**
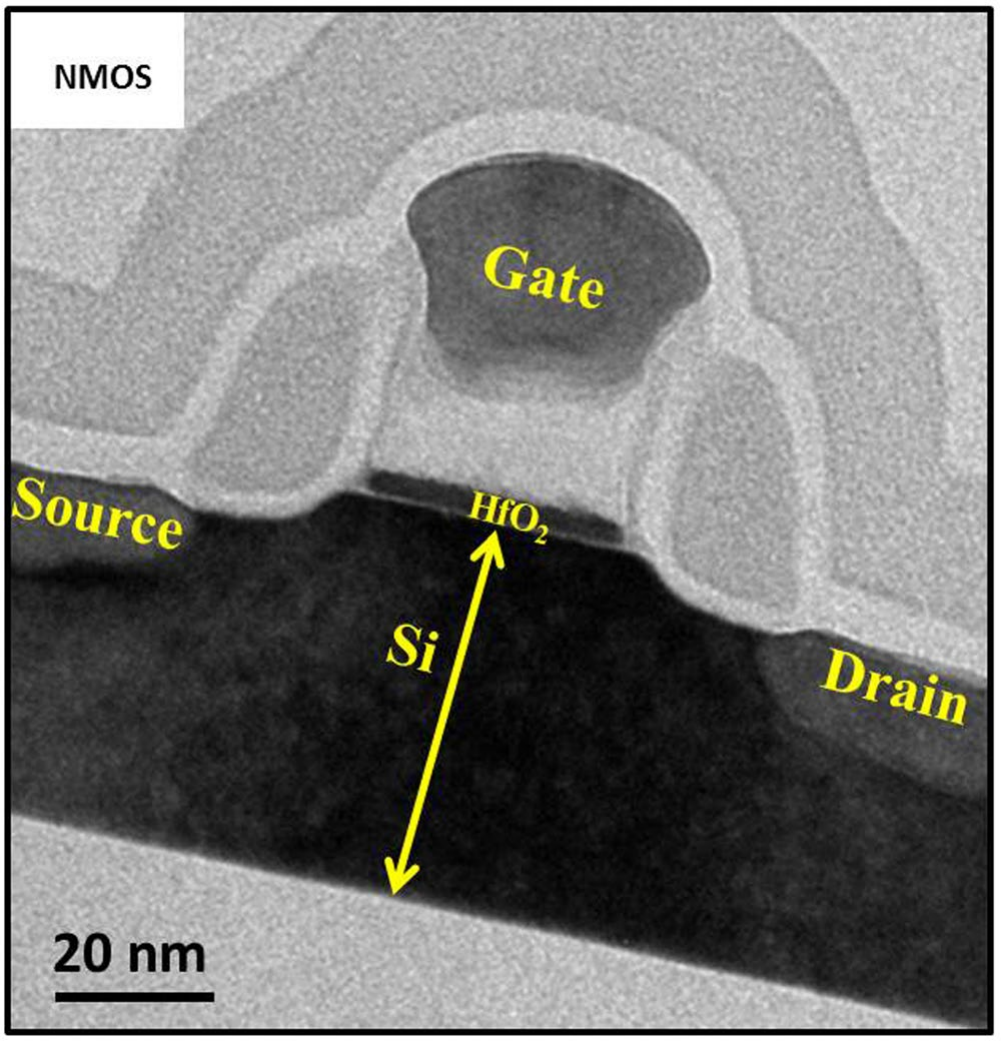
TEM image of the fabricated PD-SOI MOSFET at 32 nm technology node with 1.7 nm HfO_2_ gate oxide.

The devices used in this study were fabricated using the 32 nm SOI High-k Metal Gate (HKMG) CMOS technology^25, 26^. The gate dielectric stack is composed of 1.7 nm HfO_2_ (ALD) and 0.8 nm interfacial SiO_2_ layer which is chemically grown. Lanthanum is used as the capping layer between HfO_2_ and TiN metal gate for *V*_*T*_ adjust. Excellent CMOS performance and manufacturability is demonstrated earlier^27^. Figure 6 shows TEM image of the fabricated PD-SOI MOSFET at 32 nm technology node. The devices of 100 nm channel length and 1μm channel width are characterized in DC and transient modes by Keysight B1500 DC and Waveform Generation and Fast Measurement Unit (WGFMU) system. All the measurements are performed at room temperature.

## Electronic supplementary material

Supplementary Information

## References

[1] Merolla, P. *et al*. A Digital Neurosynaptic Core Using Embedded Crossbar Memory with 45pJ per Spike in 45 nm. IEEE Custom Integrated Circuits Conference (CICC). 1–4 (2011).

[2] Shin J, Koch C (2003). Dynamic Range and Sensitivity Adaptation in a Silicon Spiking Neuron. 1232 IEEE Trans. On Neural Networks..

[3] Hynna, K. M. & Boahen, K. Silicon neurons that burst when primed. *2007 IEEE Int*. *Symp*. *Circuits Syst*. 3363–3366, doi:10.1109/ISCAS.2007.378288 (2007).

[4] Hynna, K. M. & Boahen, K. Neuronal Ion-Channel Dynamics in Silicon the oppositeeffct: nneabydifsiongi. 3614–3617 (2006).

[5] Joubert, A., Belhadj, B., Temam, O. & Heliot, R. Hardware spiking neurons design: Analog or digital? *2012 Int*. *Jt*. *Conf*. *Neural Networks* 1–5, doi:10.1109/IJCNN.2012.6252600 (2012).

[6] Joubert, A., Belhadj, B. & Héliot, R. A robust and compact 65 nm LIF analog neuron for computational purposes. *2011 IEEE 9th Int*. *New Circuits Syst*. *Conf*. *NEWCAS 2011*, 9–12, doi:10.1109/NEWCAS.2011.5981206 (2011).

[7] Basu A, Shuo S, Zhou H, Hiot Lim M, Huang G-B (2012). Silicon spiking neurons for hardware implementation of extreme learning machines. Neurocomputing.

[8] Ostwal V, Meshram R, Rajendran B, Ganguly U (2015). An ultra-compact and low power neuron based on SOI platform. Int. Symp. VLSI Technol. Syst. Appl. Proc..

[9] Das, B., Sushama, S. & Ganguly, U. Sub-0. 2 V Impact Ionization in Si n-i-p-i-n Diode. **63**, 4668–4673 (2016).

[10] Maass, W. & Bishop, C. M. Pulsed Neural Networks. MIT Press, Massachusetts **275** (1999).

[11] Hodgkin AL, Huxley AF (1990). A quantitative description of membrane current and its application to conduction and excitation in nerve. Bull. Math. Biol..

[12] Izhikevich EM (2003). Simple Model of Spiking Neurons. Neural Networks.

[13] Borisyuk M, Borisyuk GN (1997). Neuronal Activity. Sci. York.

[14] Schlotterer, H. Substrate and the Finite Volume. *IEEE Trans*. *Electron Devices* (1975).

[15] Rajendran B (2013). Specifications of nanoscale devices and circuits for neuromorphic computational systems. IEEE Trans. Electron Devices.

[16] Indiveri G, Chicca E, Douglas R (2006). A VLSI Array of Low-Power Spiking Neurons and Bistable Synapses With Spike-Timing Dependent Plasticity. Ieee Trans. Neural Networks.

[17] Wijekoon JHB, Dudek P (2008). Compact silicon neuron circuit with spiking and bursting behaviour. Neural Networks.

[18] Moon, K. *et al*. ReRAM-based analog synapse and IMT neuron device for neuromorphic system. *2016 Int*. *Symp*. *VLSI Technol*. *Syst*. *Appl*. *VLSI-TSA 2016*, 9–10, doi:10.1109/VLSI-TSA.2016.7480499 (2016).

[19] Park, J. *et al*. 3D-stacked TiO x -based analog synapse and oscillator neuron for neuromorphic image recognition system with hardware-based edge detection capability. 4–7 (2016).

[20] Tuma T, Pantazi A, Le Gallo M, Sebastian A, Eleftheriou E (2016). Stochastic phase-change neurons. Nat. Nanotechnol..

[21] Shukla, A., Kumar, V. & Ganguly, U. A Software-equivalent SNN Hardware using RRAM array for Asynchronous Real-time Learning. **1** (2017).

[22] Hodgkin, A. L. & Huxley, A. F. *J. Physiol.* **117**, 500–544 (1952). 10.1113/jphysiol.1952.sp004764 PMC1392413 12991237

[23] Purves, D. *et al*. Neuroscience (ed. Purves, D.) 773 (Sinauer Associates, 2004).

[24] Rajendran B (2013). Specifications of Nanoscale Devices and Circuits for Neuromorphic Computational Systems. IEEE Trans. Electron Devices.

[25] Butt, N., Mcstay, K. & Cestero, A. A 0.039 um 2 high performance eDRAM cell based on 32 nm High-K/Metal SOI technology. *IEEE Internation l Electron Devices Meeting*. 616–619 (2010).

[26] Greene, B. *et al*. High performance 32 nm SOI CMOS with high-k/metal gate and 0.149mm^2^ SRAM and ultra low-k back end with eleven levels of copper. *2009 Symp*. *VLSI Technol*. 140–141 (2009).

[27] Horstmann M (2009). Advanced SOI CMOS transistor technologies for high-performance microprocessor applications. Proc. Cust. Integr. Circuits Conf..

