# Supplementary material for: Leaky Integrate and Fire Neuron by Charge-Discharge Dynamics in Floating-Body MOSFET
Source: Sci Rep. 2017 Aug 15;7:8257. doi: 10.1038/s41598-017-07418-y (PMC5557947; doi:10.1038/s41598-017-07418-y)
Supplement: Supplementary file 1 — Supplementary Information [file 41598_2017_7418_MOESM1_ESM.pdf]

Supplementary Information

Leaky Integrate and Fire Neuron by Charge-Discharge Dynamics in Floating-Body MOSFET

Sangya Dutta[1*], Vinay Kumar[1], Aditya Shukla[1] , Nihar R. Mohapatra[2], and Udayan Ganguly[1†]

[1]*Department of Electrical Engineering, IIT Bombay, Mumbai 400076, India*
[2]*Department of Electrical Engineering, IIT Gandhinagar, Gandhinagar 382355, India*

**sangya@ee.iitb.ac.in; [†]udayan@ee.iitb.ac.in*

Supplementary Information 1:

Circuit realization of the SOI neuron with an estimation of area and power:

Figure S1 shows the circuit diagram for the neuron, connected at the output of a synaptic crossbar array. The crossbar array is assumed to take positive voltages V_i as input across synapses and it sums up all the synaptic currents which are proportional to the synapse weights and gives current to the neuron input as $I_{in} \propto \sum w_i V_i$ where w_i are synaptic weights. The neuron uses the proposed SOI device (M1) in a configuration where the source of the SOI device takes voltage as input, gate is grounded and drain is used for resetting the neuron. The rest of the neuron has four stages described in detail next.

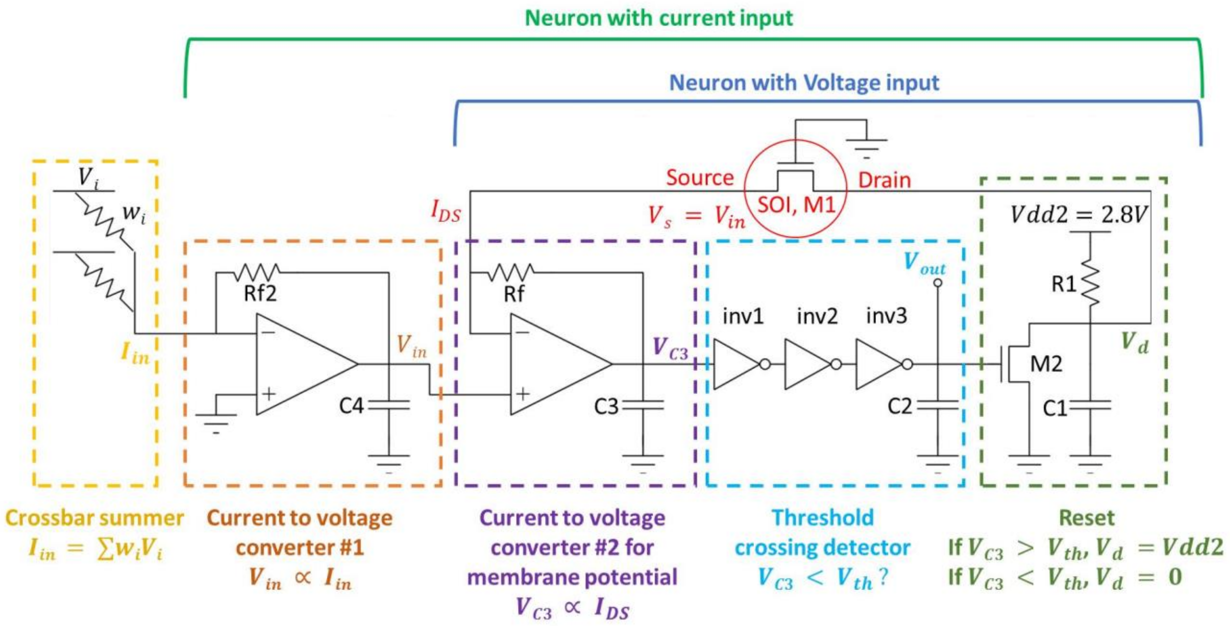

Fig. S1: Circuit diagram for SOI neuron

1. **Current to voltage converter #1:**

   As the SOI device takes voltage as input, the current I_{in} has first to be converted to a proportional voltage. So, I_{in} is fed to an OpAmp which acts as a current to voltage converter and it also provides ground to the synapse array. The voltage thus obtained is proportional to I_{in} and is used as V_{in} to the next stage.

2. **Current to voltage converter #2, membrane potential:**

   This second OpAmp takes V_{in} (a negative voltage) from previous stage and applies to the source of SOI device through virtual short. It also converts drain to source current (I_{DS})

of the SOI device into a proportional voltage that can be used to reset the neuron when it crosses a threshold. Thus, output of this stage, V_{C3} acts as membrane potential of the neuron. V_{C3} swings between zero and Vss where Vss is the negative rail voltage applied to the OpAmp along with positive rail voltage Vdd.

3. **Threshold crossing detector:**

This stage has three inverters which charge a capacitor C2 if membrane potential $V_{C3} < V_{th}$ and discharge if $V_{C3} > V_{th}$. The first inverter converts the swing of membrane potential from negative to positive between zero and Vdd. The second inverter sharpens the response of first inverter. The third inverter has a fast pull up and a slow pull down so that C2 is charged up as soon as membrane potential goes below V_{th} providing immediate reset to the neuron and also ensuring that membrane potential does not start charging again before I_{DS} resets completely so that the whole process can be repeated. Voltage across C2 can be used as positive voltage spikes, V_{out}.

4. **Reset by SOI drain voltage control:**

This stage charges drain voltage (V_d) of SOI towards $Vdd2 = 2.8V$, through a resistance R1 and capacitance C1. R1 is small as compared to 'on' resistance of SOI. So, as V_d approaches $Vdd2$, I_{DS} increases sharply due to impact ionization and hole storage in potential well as described earlier. When I_{DS} is high enough, V_{C3} falls below threshold and V_{out} turns M2 on which shorts C1 and hence V_d is set to zero. As a result, I_{DS} and V_{out} also go to zero thus resetting the neuron. As C2 voltage falls below threshold voltage of M2, C1 starts charging again and V_d starts to rise again, repeating the whole process.

Figure S2 shows the output characteristics of the neuron using the above mentioned reset circuit. The spiking behavior is shown here for $V_{in} = -0.3\ V$.

Figure S3 shows the detailed circuits for OpAmp and inverter chain along with the layouts of all stages. The OpAmps are similar so only one layout has been shown. Capacitors C2, C3, and Cc along with resistance Rf, and some Metal-2 and higher connections have not been shown in the layouts to maintain clarity of figures.

The total area for neuron with current input, including all capacitances (Capacitor density of 15fF/0.039μm^2 using Deep Trench technology[1] at 32nm technology node, has been estimated to be 2.33μm^2 or 2275.4 F^2. The energy consumed to generate one spike is 54pJ. To compare with (*phase change and other neurons*) which are voltage input neurons, we can consider the neuron with voltage as input (Figure S1) which does not have the first OpAmp stage of neuron described previously. This voltage input neuron has an area of 1.81μm^2 or 1767.6 F^2 and energy consumed per spike is 35 pJ.

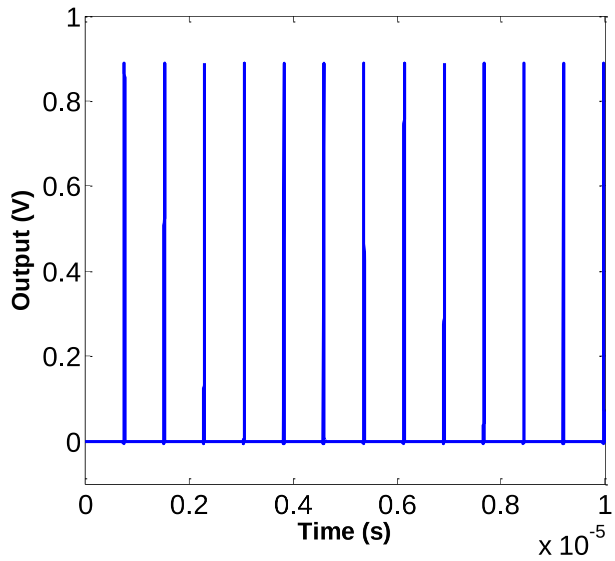

Fig. S2: Output of the reset circuit showing spiking at $V_{in} = -0.3V$

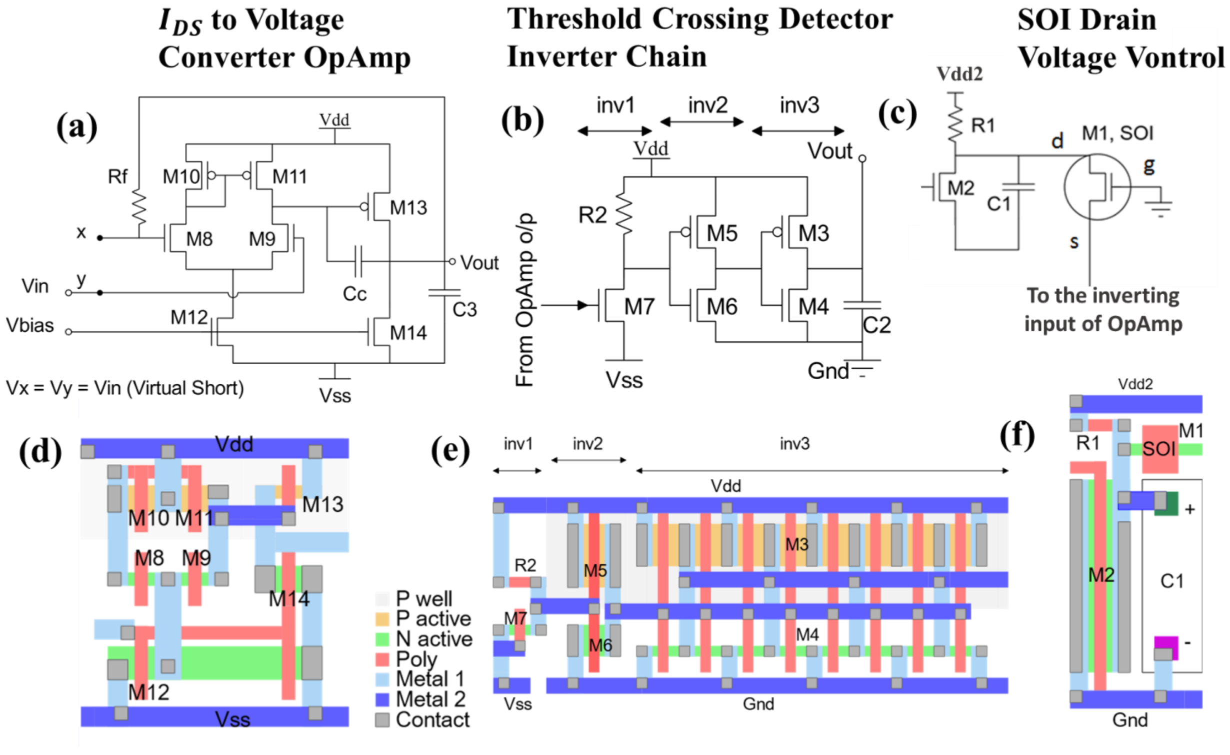

Fig. S3: Layout shown separately for each section of the proposed reset circuit. (a),(d): Op-Amp internal circuitry and the layout design. (b),(e): Circuit design to realize the inverter chain using MOS transistors and the layout. (c),(f): The SOI neuron with the charging-discharging unit and the layout. Capacitors $C2$, C3, and Cc along with resistance Rf and some Metal and higher connections have not been shown in the layouts to maintain clarity of figures.

Supplementary Information 2:

Performance Evaluation of SOI Neuron in a Spiking Neural Network:

The spiking frequency of the device, as a function of DC input bias is plotted in figure S4. We mathematically implement a LIF neuron model (as shown in figure 2(a) in the original manuscript) such that the model response (solid curve) represents the experimental f-I curve with excellent match. Such an implementation enables an SNN study. Next, a typical SNN classifier for Fisher's iris dataset in MATLAB is used as follows[2]. 12 LIF input neurons are connected to 3 output neurons out through a 12×3 synaptic array (figure S5). The drop in recognition error as a function of training iterations is plotted in Fig. S6. 95% accuracy is achieved for the Fisher Iris classification dataset, which matches the state of the art for SNN[2,3].

To convert such an algorithm to an asynchronous spiking neural network *hardware*, we have proposed a two-array scheme explained in details elsewhere[4]. Essentially, two physically separated same-sized resistive-RAM (RRAM) arrays have been used to resolve the "write-read dilemma" in implementation of asynchronous SNN in cross-bar arrays (see figure S7). One array is for recognition (read) operations, while the other is for performing learning (write) operations. The learning occurs by synaptic-time-dependent plasticity (STDP) rule by pre- and post-neuronal waveform superposition[5], which has been demonstrated experimentally[6]. Periodically, we copy the write array weights to the read array. Thus, as learning progresses (or equivalently, weights evolve), recognition improves. With such a scheme, we have shown software equivalence in terms weight evolution, signal processing equivalence, and learning performance. Thus, the software algorithm above can be converted to hardware implementation.

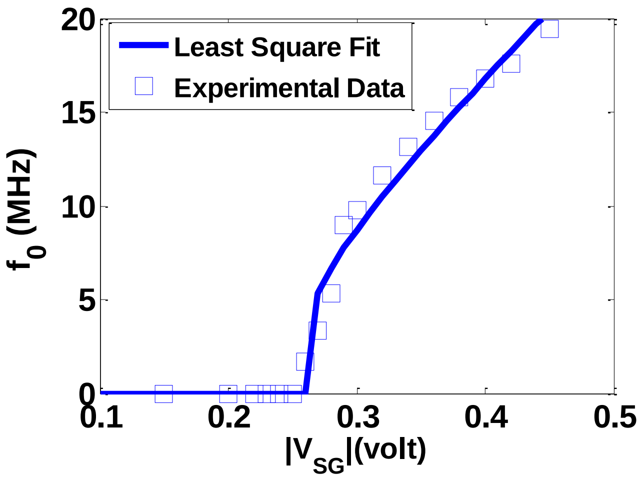

Fig. S4: Output characteristics of SOI neuron. Experimental frequency vs. input voltage shows thresholding at $|V_{SG}| = 0.26\,V$ (squares). The solid line is the equivalent LIF neuron model (parallel RC circuit).

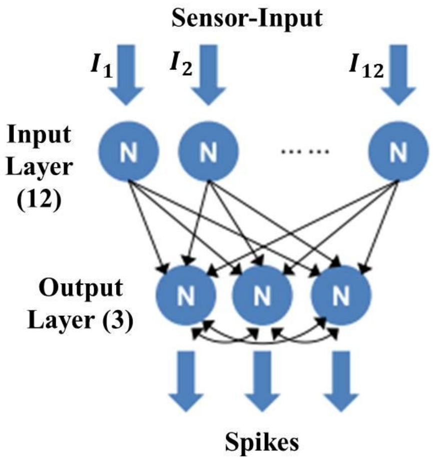

Fig. S5: Signal flow diagram in a (12×3) array[1].

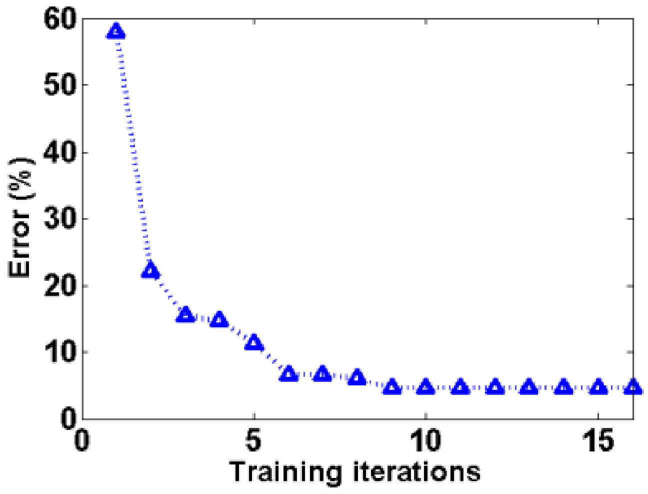

Fig. S6: Recognition task is being performed using Fisher's Iris database in MATLAB. Simulated performance shows increasing accuracy with training iterations.

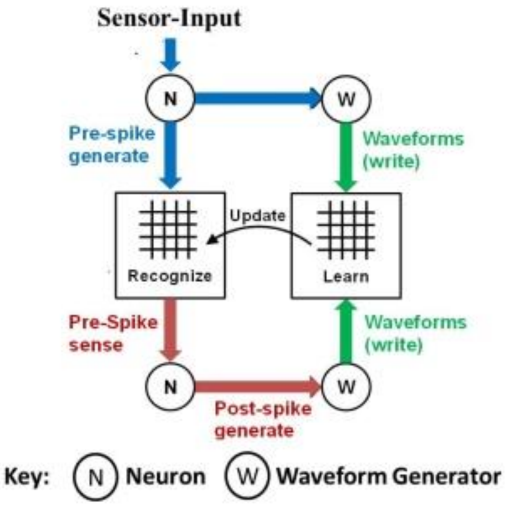

Fig. S7: Two array spiking neural network where recognition and learning is done in separate arrays simultaneously[4].

References

1. Wang, G. *et al.* Scaling deep trench based eDRAM on SOI to 32nm and beyond. *Tech. Dig. - Int. Electron Devices Meet. IEDM* 259–262 (2009). doi:10.1109/IEDM.2009.5424375

2. Biswas[o], A., Prasad, S., Lashkare, S. & Ganguly, U. A simple and efficient SNN and its performance & robustness evaluation method to enable hardware implementation.

3. Xin, J. & Embrechts, M. J. Supervised learning with spiking neural networks. *Int. Jt. Conf. Neural Networks. Proc. (Cat. No.01CH37222)* **3,** 1772–1777 (2001).

4. Shukla, A., Kumar, V. & Ganguly, U. A Software-equivalent SNN Hardware using RRAM array for Asynchronous Real-time Learning. **1,**

5. Rajendran, B. Specifications of Nanoscale Devices and Circuits for Neuromorphic Computational Systems. *IEEE Trans. Electron Devices* **60,** 246–253 (2013).

6. Panwar, N., Rajendran, B. & Ganguly, U. Arbitrary Spike Time Dependent Plasticity (STDP) in Memristor by Analog Waveform Engineering. *IEEE Electron Device Lett.* **3106,** 1–1 (2017).
